# Broadband Silicon Nitride Power Splitter Based on Bent Directional Couplers with Low Thermal Sensitivity

**DOI:** 10.3390/mi13040559

**Published:** 2022-03-31

**Authors:** Donghao Li, Bin Li, Bo Tang, Peng Zhang, Yan Yang, Ruonan Liu, Ling Xie, Zhihua Li

**Affiliations:** 1 Institute of Microelectronics, Chinese Academy of Sciences, Beijing 100029, China; lidonghao@ime.ac.cn (D.L.); tangbo@ime.ac.cn (B.T.); zhangpeng1@ime.ac.cn (P.Z.); yyang10@ime.ac.cn (Y.Y.); liuruonan@ime.ac.cn (R.L.); xieling@ime.ac.cn (L.X.); 2 University of Chinese Academy of Sciences, Beijing 100049, China

**Keywords:** directional couplers, silicon nitride, silicon photonics, bent waveguides

## Abstract

Directional couplers, as power splitters, have provided a significant contribution for light splitting and combining in silicon photonics. However, the splitting ratio of conventional directional couplers is very sensitive to wavelength, which limits the bandwidth and the transmission performance of the devices. In this work, a silicon nitride bent directional coupler with large bandwidth, large fabrication tolerance, and low thermal sensitivity is proposed and demonstrated through simulation analysis and experiments. Moreover, the fabrication process of 400 nm thick silicon nitride photonic devices is described, which are compatible with complementary metal–oxide–semiconductor technology. The 1 dB bandwidth of the bent waveguide coupler can reach 80 nm, and the thermal sensitivity is reduced by 85% compared to the silicon-based devices.

## 1. Introduction

Silicon-based optical power splitters are essential devices for large-scale photonic integration. Directional couplers (DC) have been widely used as power splitters in optical communications, optical computing, laser radar, and other fields [1,2,3,4,5], due to their simple structure and easy manufacturing. The bottleneck issue in using DC is the wavelength-sensitive and fabrication-sensitive splitting ratio [6], which requires precise control over the fabrication variation and input optical wavelength. Therefore, the conventional DC is not suitable for applications with a wide range of wavelengths. Many different approaches have been proposed in order to develop broadband power splitters. Hybrid plasmonic couplers with plasmonic metal deposition can increase operational bandwidth, but at the expense of increased manufacturing complexity and cost [7]. Asymmetric DCs can reduce the wavelength sensitivity by designing two waveguides with different widths in the coupling region, but this coupler has high sensitivity to waveguide width deviation [8]. Subwavelength gratings (SWG) can be used to enhance the bandwidth of DCs without affecting the device size. However, SWG directional couplers are sensitive to grating pitch and fill-factor variations [9]. Moreover, theoretical research and simulation results indicate that DCs with bent waveguides can realize wavelength insensitivity, a small footprint, and tolerance to fabrication [10].

Compared with silicon, silicon nitride is more suitable for the manufacture of low-loss photonic devices and compatibility with complementary metal–oxide–semiconductor (CMOS) processes [11]. Silicon nitride has a moderate index contrast, which relaxes fabrication tolerance and reduces the sidewall roughness scattering [12]. In addition, the thermo-optical coefficient of silicon nitride (~2.45 × 10^−5^/°C) is one order of magnitude smaller than that of silicon (~1.86 × 10^−4^/°C); thus, silicon nitride devices have less temperature sensitivity [13]. All these properties make silicon nitride an ideal material for use in passive photonic devices.

In this work, a DC with bent waveguides on a silicon nitride platform is demonstrated numerically and experimentally. Firstly, the working principles of the bent DC are explained thoroughly, and a simulation analysis is performed. Then, the fabrication process and the measurement of the devices are described in detail. The results show that the silicon nitride DC with bent waveguides achieved a 1 dB bandwidth of 80 nm centered at 1550 nm, which is about twice as much as a conventional DC. Moreover, the bent DC could still maintain good performance under the condition of temperature fluctuation and fabrication variation.

## 2. Working Principle and Design Structure

**Figure 1:**
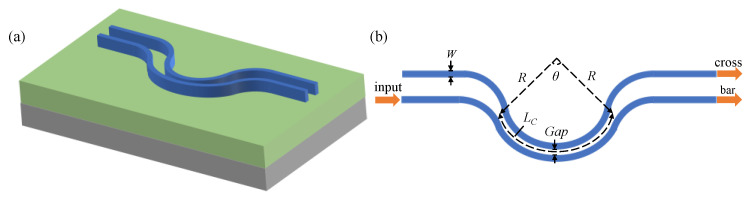
Schematic structure of the bent DC: (**a**) 3D sketch; (**b**) top view and design parameters.

The schematic structure of the bent DC is shown in Figure 1, which consists of two adjacent waveguides. The part of parallel bent waveguides is the coupling region, in which the mode field in one waveguide is coupled to the other waveguide through the evanescent field. *W* is the width of the waveguide, *Gap* is the separation of the waveguide in the coupling region, *R* and *θ* are the bending radius and angle of the bent waveguide, and *L_C_* is the length of the coupling region, which is determined by $L_{C}=\theta \cdot R$. This means that the length of the coupling region can be adjusted by *R* and *θ*, thus changing the splitting ratio of the bent DC.

According to the coupled mode theory [14], the output power of a DC is given by

(1) $$\left\{ \begin{array}{l} P_{1}=P_{0}-P_{0}{\left(\frac{\kappa_{C}}{\gamma }\right)}^{2}\sin^{2}\left(\gamma L_{C}\right)\\ P_{2}=P_{0}{\left(\frac{\kappa_{C}}{\gamma }\right)}^{2}\sin^{2}\left(\gamma L_{C}\right) \end{array}\right.,$$

where *P*_0_ is the input power, *P*_1_ and *P*_2_ are the output power of bar port and cross port, *L_C_* is the length of the coupled waveguide, *κ_c_* is the coupling coefficient, and *γ* is given by

(2) $$\gamma =\sqrt{{\left(\frac{\beta_{1}-\beta_{2}}{2}\right)}^{2}+\kappa_{{}^{C}}^{2}},$$

where *β*_1_ and *β*_2_ are the propagation constants of two waveguides, which are determined by the effective refractive indices of waveguides. The amplitude $\frac{\kappa_{C}}{\gamma }$ determines the maximum coupling efficiency between two parallel bent waveguides. In the bent waveguide, the effective refractive index of the waveguide changes with the bending radius [15]. Thus, the propagation constants of the two parallel waveguides are different, which means that $\beta_{1}\neq \beta_{2}$ and the amplitude $\frac{\kappa_{C}}{\gamma }$ is less than 100%. With the change in wavelength, the variation of the splitting ratio decreases, thus realizing wavelength insensitivity.

**Figure 2:**
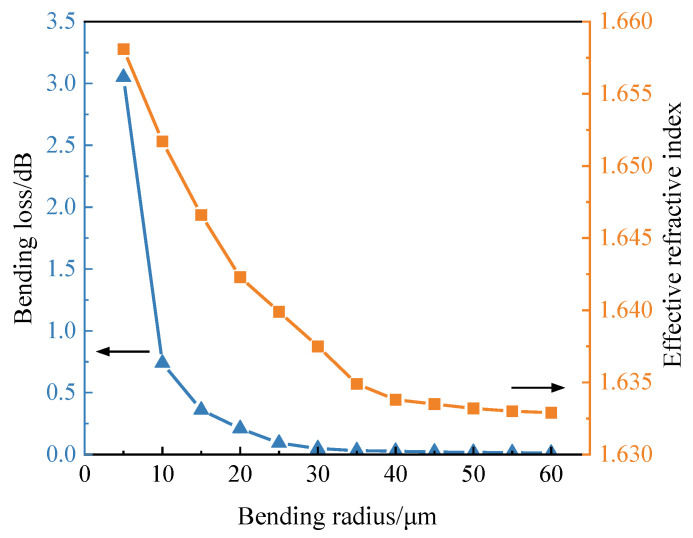
Relationship among effective refractive index, bending loss, and bending radius of 1 μm wide and 400 nm thick silicon nitride waveguide.

The whole device is based on a 400 nm thick silicon nitride waveguide layer with a 3 μm thick layer of buried silicon dioxide, which can isolate the waveguides from the substrate to reduce the loss due to the light leakage from the substrate. The three-dimensional finite difference time domain (FDTD) method was used to simulate the bent DC. The refractive indices of silicon nitride and silicon dioxide at the wavelength of 1550 nm are 1.98 and 1.445. The width of the waveguide (*W*) was fixed to 1 μm, and the spacing between the two waveguides (*Gap*) was designed as 300 nm. The relationship among effective refractive index, loss, and bending radius (*R*) of the bent waveguide is shown in Figure 2. As the bending radius decreases, the effective refractive index increases, resulting in a flattening of the splitting ratio. However, a small bending radius would bring about large bending loss, due to the mismatch of the mode between bent and straight sections of the waveguide [16].

**Figure 3:**
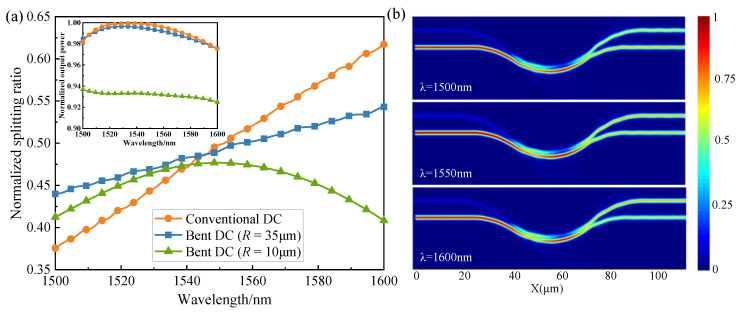
(**a**) Wavelength dependence of transmittance of the different DC at cross port; inset: the total output power of the different DCs; (**b**) 3D FDTD simulated power distribution of bent DC with *R* = 35 μm and *θ* = 25°.

The simulated results of different bending radii of the bent DC and the conventional DC are shown in Figure 3a. The transmittances of the bar port and cross port obtained by the FDTD method indicate the normalized splitting ratio of the device, and the normalized output power of the device can be calculated by adding the transmission of the two ports. Within the 100 nm wavelength range from 1500 nm to 1600 nm, the deviations of the splitting ratio (cross port) of the three devices are 24%, 9%, and 6%, respectively. The bent DC with 10 μm bending radius exhibits the most flattened transmission, but the loss of this device reaches 0.3 dB, as shown in the inset, which cannot satisfy the demand of low-loss photonic integration. The bent DC with 35 μm bending radius has almost no extra bending loss and achieves nearly a threefold enhancement in bandwidth compared with the conventional DC. Thus, the bending radius (*R*) and angle (*θ*) of the bent DC were selected to be 35 μm and 25°, respectively. The optical power distributions of the designed bent DC at different wavelengths are shown in Figure 3b. The optical power distributions are almost the same at different wavelengths, indicating that the device is wavelength-insensitive. Therefore, the bent DC exhibits low loss, large bandwidth, and wavelength-insensitive characteristics.

## 3. Fabrication and Characterization

### 3.1. Fabrication Process

The bent DC was fabricated on the silicon nitride platform, which is compatible with CMOS processes. Silicon nitride films are usually deposited by low-pressure chemical vapor deposition (LPCVD) at high temperature (>700 °C) and plasma-enhanced chemical vapor deposition (PECVD) at low temperature (<400 °C) [13]. The silicon nitride films deposited by LPCVD have less particle pollution, exhibit high uniformity of thickness, and are close to stoichiometric Si_3_N_4_. However, LPCVD-based silicon nitride films which are directly deposited on the wafer will produce a large tensile stress [17]. If the stress exceeds the strength of the silicon nitride films, the wafer will crack. In this work, high-quality 400 nm thick silicon nitride films were obtained through deposition and annealing in two steps, which can effectively solve the problem of stress in the deposition [18].

**Figure 4:**
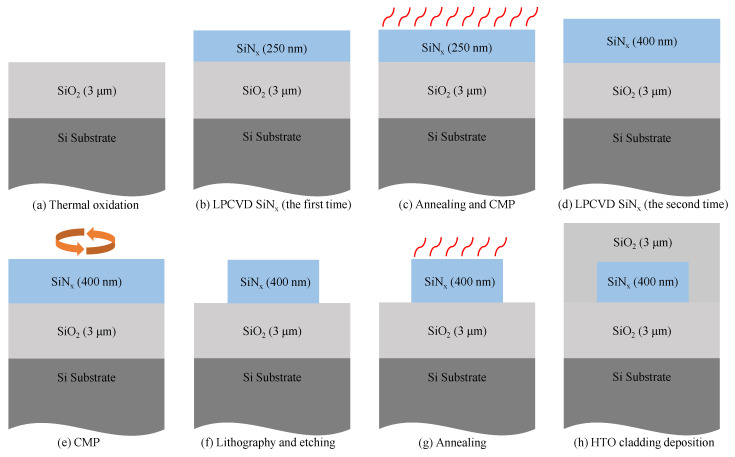
Process flow for cross-section of 400 nm thick silicon nitride films.

The fabrication started with the 3 μm thick thermal oxidation of the silicon substrate on the 200 mm wafer, as shown in Figure 4. Firstly, 250 nm thick silicon nitride films were deposited by LPCVD and annealed at 1050 °C for 9 h to reduce stress. After annealing, chemical mechanical polish (CMP) was used to planarize the surface of the films. Then, the second deposition of 200 nm thick silicon nitride films was carried out, followed by lithography and etching to obtain the structure of the device, and this step could further release the stress of the film. Finally, the second annealing was performed, and 3 μm thick silicon dioxide was deposited as the upper cladding.

**Figure 5:**
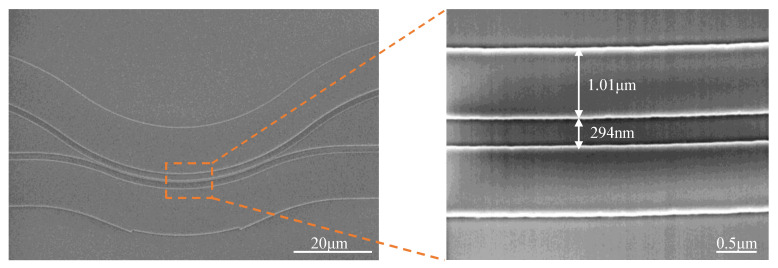
SEM image of the bent DC.

The top-view scanning electron microscope (SEM) image of the bent DC is shown in Figure 5. The measured width of the bent waveguide was 1.01 μm (versus a designed width of 1 μm), and the actual width of *Gap* was 294 nm (versus a designed width of 300 nm), satisfying the requirements for the devices.

### 3.2. Characterization of the Bent Directional Coupler

**Figure 6:**
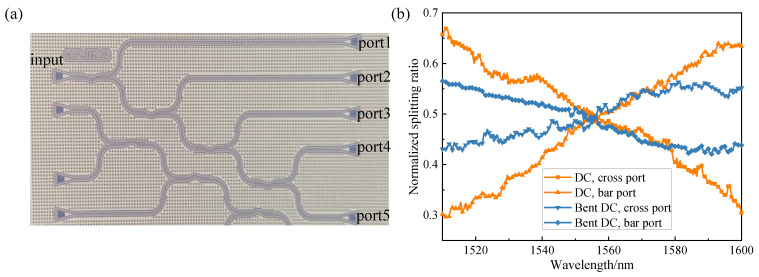
(**a**) The test structure of the bent DC; (**b**) the measured transmission spectra of the conventional DC and the bent DC.

A tunable laser was applied as the input light source, and transverse electric (TE) polarized light was obtained utilizing an optical polarizer and a polarization controller. The laser source was coupled to the input port of the bent DC through a focusing grating coupler. The transmitted light from the cross port and bar port was coupled out through another grating coupler and collected by a fiber connected to the optical spectrum analyzer or optical power meter. The bent DC adopted the cascade test structure as shown in Figure 6a. The insertion loss of the device could be obtained by the slope of the fitting curve for the output power of ports 1–4. The normalized splitting ratio could be obtained by measuring the output spectra of port 4 and port 5, followed by normalization. Normalization refers to the conversion of the output power value (dB) into a percentage, so that the change in the splitting ratio of the device can be observed more intuitively.

The measured transmission spectra of the conventional DC and the bent DC are shown in Figure 6b. In the wavelength range from 1510 nm to 1600 nm, the splitting ratio of the cross port and bar port was between 0.3 and 0.65 in the conventional DC, while the splitting ratio of the two ports fluctuated within ±8% in the bent DC. Compared with the 1 dB bandwidth of 40 nm in the conventional DC, the 1 dB bandwidth of the bent DC could reach 80 nm, and the insertion loss was approximately 0.25 dB. The bent DC has the advantages of a large bandwidth and wavelength insensitivity, and it can meet the requirements of wavelength division multiplex (WDM) systems, which are important in optical communication and optical interconnection [19].

The performance of the device is quite different in theoretical design and experimental results due to the fabrication variation, and the characteristic deviation of a single device will affect the operation of the whole optical system [20]. Considering that the fabrication variation generally fluctuates within 10%, the *Gap* of the bent DC was selected as 270 nm, 300 nm, and 330 nm, and the width of the waveguide (*W*) was set as 1.03 μm, 1 μm, and 0.97 μm.

**Figure 7:**
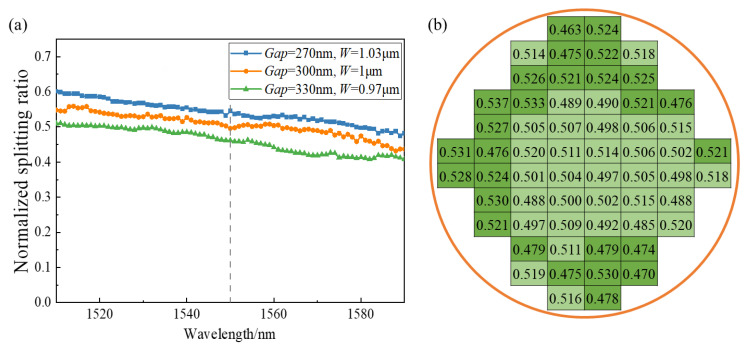
(**a**) Wavelength dependence of transmission of bent DC (cross port) with different sizes; (**b**) wafer-level measurements of splitting ratio at cross port of bent DC.

As shown in Figure 7a, the splitting ratio (cross port) changed within ±3% (the relative change was ±6%) at the wavelength of 1550 nm when the width of the bent DC changed within ±30 nm (the relative change was ±10%), while the splitting ratio of the conventional DC varied from −3 dB to −2.4 dB as the *Gap* decreased by 30 nm [10], indicating that the bent DC has better fabrication tolerance. On the one hand, with the decrease in waveguide width (*W*), the confinement of the light field became weaker, and the evanescent field distribution increased, which enhanced the coupling with another waveguide (the coupling coefficient *κ_c_* increased). On the other hand, the *Gap* became larger as the waveguide width (*W*) decreased, which weakened the coupling between the two waveguides (the coupling coefficient *κ_c_* decreased). Thus, the bent DC was insensitive to fabrication variation. The wafer-level measurement results in Figure 7b demonstrate that the splitting ratio of half the devices on the wafer fluctuated within ±2% (light green), while all devices varied within ±4% (dark green).

**Figure 8:**
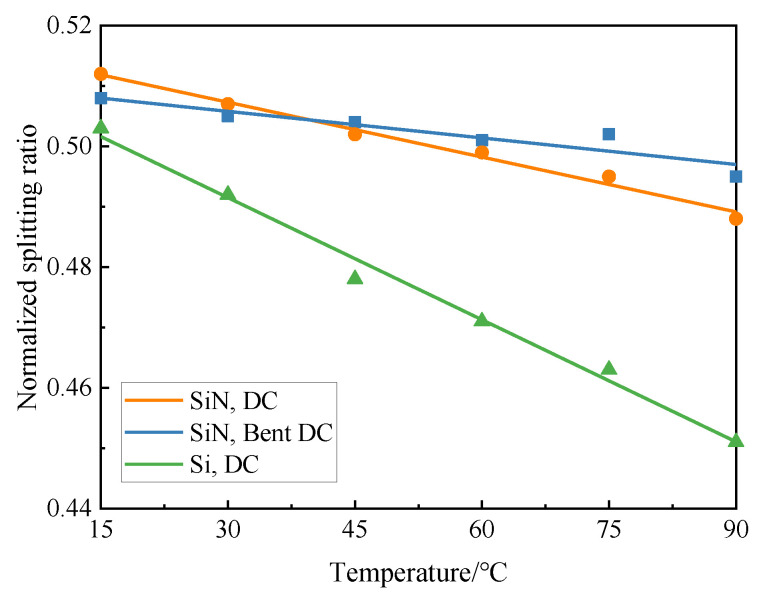
Temperature dependence of splitting ratio (cross port) of the silicon-based conventional DC, silicon nitride conventional DC, and silicon nitride bent DC at the wavelength of 1550 nm.

The coupling coefficient of DC changes with the fluctuation of temperature because of the variation in the refractive index of materials caused by the thermo-optical effect. This affects the splitting ratio and results in a degradation of device performance. The relationship between temperature and splitting ratio (cross port) of the silicon conventional DC, silicon nitride conventional DC, and silicon nitride bent DC at the wavelength of 1550 nm is shown in Figure 8. It can be found that, as the temperature increased from 15 °C to 90 °C, the splitting ratio of silicon-based DC varied between 0.45 and 0.5, whereas the silicon nitride conventional DC varied its splitting ratio by approximately ±1% and the bent DC changed its splitting ratio by less than ±0.6%. Compared to the silicon-based devices, the silicon nitride bent DC achieved a reduction in thermal sensitivities of about 85% and presented low thermal sensitivity, indicating its adaptability to complex temperature conditions.

## 4. Conclusions

A bent DC was designed and fabricated on a 400 nm thick silicon nitride platform. The experimental results suggest that the bent DC obtained a 1 dB bandwidth of 80 nm, which was about twice that of the conventional DC. A fabrication variation of 10% was considered, and the bent DC proved to be fabrication-tolerant in terms of waveguide width and separation in the coupling section. When the temperature changed within 75 °C, the splitting ratio of the bent DC varied less than ±0.6%. Compared with silicon-based devices, the silicon nitride bent DC reduced temperature sensitivity by 85%. Furthermore, the splitting ratio of bent DC could be adjusted by changing the bending radius (*R*) and angle (*θ*) of the bent waveguide, meeting different application requirements. The research on silicon nitride power splitters can pave the way for high-performance large-scale photonic integration.
